# A Clinical Overview of Anorexia Nervosa and Overcoming Treatment Resistance

**DOI:** 10.1055/s-0042-1758859

**Published:** 2022-12-21

**Authors:** Hassan Nagy, Tanya Paul, Esha Jain, Hanyou Loh, Syeda Hafsa Kazmi, Rishbha Dua, Ricardo Rodriguez, Syed Ali Abbas Naqvi, Metu Chiamaka U., Erjola Bidika

**Affiliations:** 1 Division of Research & Academic Affairs, Larkin Health System, South, Miami, Florida, United States

**Keywords:** anorexia nervosa, treatment-resistant anorexia, eating disorders, psychiatric disorders, managing chronic anorexia, psychosocial treatments of anorexia, deep brain stimulation in anorexics

## Abstract

Anorexia nervosa (AN) is a type of eating disorder that has been increasing in incidence and has been encountered more commonly by physicians in their daily practice. Both environmental and genetic risk factors paired along with a more susceptible neurobiology are at play in the emerging resistance to treatment in AN. Preoccupations with intense fear of weight gain, dietary restrictions, excessive exercise, and how the individual is perceived by society mixed with underlying psychopathology all further add to the issue. Many patients who fall into this cycle of obsessive and restrictive patterns refuse to get treatment. As clinicians, it is essential we recognize the early signs of both eating disorders during the initial primary care appointments.

To review the literature on the etiology of AN, possible misdiagnosis leading to inappropriate management of this condition, and understand the treatment-resistant AN and its management. Additionally, it will explore possible reasons that contribute to the resistance to treatment, the underlying psychopathology of anorexics, its genetic predisposition, psychiatric comorbidities, identification of the early signs and symptoms, and timely prevention.

Early recognition by a physician includes a thorough history and physical examination, pertinent laboratory, and electrolyte studies, and identifying comorbid psychiatric conditions. The treatment of AN is intricate and requires a holistic approach. Treatment includes multiple modalities such as nutritional rehabilitation and psychosocial and pharmacological therapies. An interdisciplinary team of medical professionals for managing chronic AN is recommended.

## Introduction

Anorexia nervosa (AN) is a condition increasingly encountered by physicians in their daily practice. It was reported by Hoek that there has been an increase in the incidence of AN up to the early 2000s, with females aged between 15 and 24 years accounting for majority of the cases. 1 This disorder was also found to be more common among Caucasians than African Americans. 1 Similar results for AN were obtained from another observational study conducted by Petkova et al between February 1, 2015 and September 10, 2015, across the United Kingdom and Ireland. 2 Through this study, it was estimated that 37 new cases of AN were reported annually for every 100,000 females aged between 10 and 19 years, while only 3 new cases were reported for every 100,000 males of the same age. 2 Majority of these cases again involved Caucasians (92%) and prevalence was estimated to range between 0.3% and 0.6%. 2

A meta-analysis of 36 studies published between January 1966 and September 2010 was performed by Arcelus et al. This study concluded that individuals with eating disorders have significantly elevated mortality rates with the highest occurring in those with AN. 3 Moreover, they reported that the cause of death was suicide for every one in five individuals who died from AN. 3 This could possibly be related to the psychiatric comorbidities associated with AN. Salbach-Andrae et al conducted a study on 101 female adolescents who were being treated at a psychiatric unit primarily for AN. 4 About 73.3% of these patients were diagnosed with comorbidities, with mood disorders being the most common at 60.4%. 4 Anxiety disorders were next in-line at 25.7%, followed by obsessive-compulsive disorder (OCD) at 16.8%, and substance use disorder at 7.9%. 4

Given the increased incidence of this condition and high mortality rate, the purpose of this paper is to review the literature on the etiology of AN, possible misdiagnosis leading to inappropriate management of this condition and understand the treatment resistance AN and its management. Using this article, we seek both to guide future research on AN and inform fellow physicians on the best forms of management for the condition.

## Etiology of Anorexia Nervosa

AN is a multifactorial eating disorder involving a combination of predisposing and precipitating factors. Predisposing factors can be biological, psychological, or environmental and include genetics, pregnancy-related factors, childhood life-events and eating behaviors, teasing and criticism or bullying from peers, personality traits, and psychiatric comorbidities. Although there is no proven involvement of genes in anorexia, some people may have a genetic tendency toward obsessive-compulsive and other personality traits, perfectionism, and sensitivity. There is strong evidence that individuals with first-degree relatives with AN are at increased risk of developing this condition. 5 Precipitating factors include dieting, weight loss, as well as stressors from life events. 6 Dieting and weight loss are major factors that predispose to AN, by directly influencing mood changes, brain function, and the further decrease in appetite. Stressors from life events including new school, job, or home; death of a loved one; or any sudden transitions increase emotional stress significantly and put individuals at increased risk for developing AN. The risk is reported to be higher in females. 7 Previous studies have indicated that the serotonin receptor HTR2A and serotonin molecule (5-HTT) are implicated in the pathogenesis of AN. 8 Moreover, different studies that have been performed in twins suggest a shared genetic factor between AN and major depression, suicide attempts, OCD, and eating disorders. 9 10 11

**Fig. 1:**
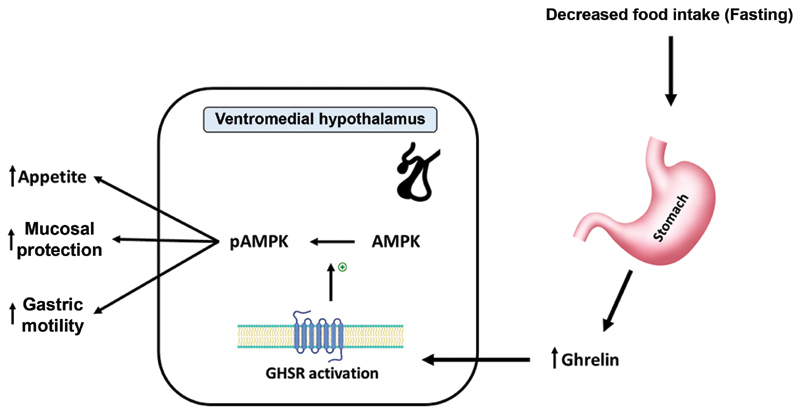
Mechanism of ghrelin in the regulation of food intake. 12 AMPK, 5′ adenosine monophosphate-activated protein kinase; GHSR, growth hormone secretagogue receptor; pAMPK, phosphorylated 5′ adenosine monophosphate-activated protein kinase.

**Fig. 2:**
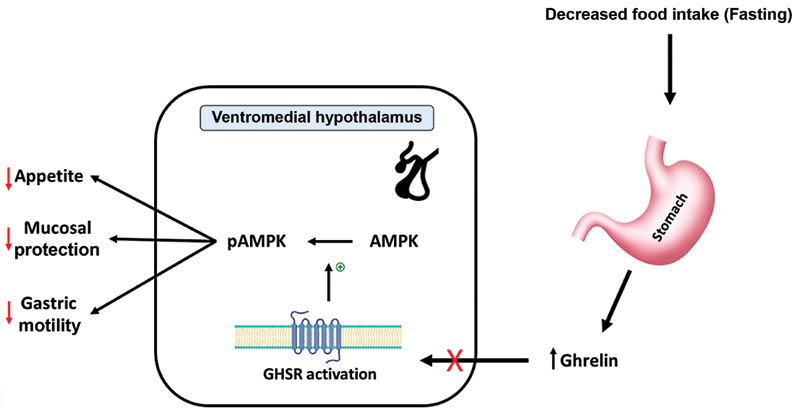
Mechanism of ghrelin resistance in the pathogenesis of anorexia nervosa. 12 (Designed by research team). AMPK, 5′ adenosine monophosphate-activated protein kinase; GHSR, growth hormone secretagogue receptor; pAMPK, phosphorylated 5′ adenosine monophosphate-activated protein kinase.

Ghrelin resistance also plays an important role in the pathogenesis of AN. 12 Ghrelin is a 28 amino-acid peptide hormone produced by the oxyntic glands in the stomach in the fasting state. As shown in Fig. 1 , the release of Ghrelin stimulates the ventromedial hypothalamus by binding and activating the growth hormone secretagogue receptor (GHSR), leading to phosphorylation of the 5′ adenosine monophosphate-activated protein kinase (AMPK), resulting in an increase in gastric motility and appetite ( Fig. 2 ). 12

## Complications of Anorexia Nervosa

Medical complications of AN can vary in the degree of severity and often they can be systemically widespread. 13 14 Common complications associated with AN include secondary amenorrhea and the presence of lanugo (thin, fine body hair). Life-threatening complications may also occur, such as those of the endocrine and cardiovascular systems, namely hypoglycemia and sudden cardiac death respectively. Additionally, other complications that may be encountered include those of the pulmonary, gastrointestinal, hematologic, dermatologic, and nervous system. 14 15 Reduced pulmonary function, aspiration pneumonia, emphysema, and spontaneous pneumothorax are potential pulmonary complications of AN. Superior mesenteric artery syndrome and gastroparesis with constipation are gastrointestinal complications that have been found to be associated with AN. 15 Gelatinous bone marrow transformation, sarcopenia, and reduced bone mass are also observed. 15 Depending on the severity of AN, anemia, leukopenia, and thrombocytopenia may also be present to varying degrees. Patients could also present with xerosis and acrocyanosis. Imaging studies of the brain have also shown evidence of atrophy, with secondary cognitive impairment manifesting clinically. 13 14 Additional complications of the endocrine system include reduced leptin levels, euthyroid sick syndrome, elevated cortisol serum levels, as well as resistance to growth hormone. Structural changes to the left ventricle and mitral valve prolapse may also be encountered. 13 14

In addition to the above medical complications of AN, complications associated with the treatment may also arise such as a refeeding syndrome (RS). 15 RS is a serious complication that can occur during the treatment of AN. This complication occurs when nutritional therapy administered to the anorexic patient is introduced in excess and can also be fatal if not managed appropriately. Hormonal and metabolic changes can occur during the refeeding of chronically malnourished patients, via either oral or parenteral methods. 15 Electrolyte derangements that may occur include hypophosphatemia, hypokalemia, hyponatremia, hypomagnesemia, and metabolic acidosis. 15 Of these, hypophosphatemia is the most important diagnostic marker for RS, and it is recommended that supplemental phosphorus be commenced early to maintain serum levels above 3.0mg/dL. 15 16 Cardiac and neurological events associated with refeeding were most frequently noted to occur within the first weeks of refeeding, making it important to closely monitor a patient's electrolyte and cardiac status during this period. 16

## History and Physical Examination of AN Patients

In order for a prompt diagnosis and successful treatment, it is crucial to recognize the clinical signs and symptoms along with the physical presentation of AN. Studies have shown that the severity of the illness AN is equivalent to lower body weights—mild: body mass index (BMI) more than or equal to 17 kg/m ^2^ , moderate: BMI 16 to 16.99 kg/m ^2^ , severe: BMI 15 to 15.99 kg/m ^2^ , extreme: BMI less than 15 kg/m ^2^ . 17 According to the Diagnostic and Statistical Manual of Mental Disorders (DSM)-IV diagnostic criteria, if a patient consistently refuses to keep their body weight equivalent to or above 85% of what is normal for their age and height, one must consider AN. 18 Concern is raised when low body weight is associated with worsened bradycardia and hypotension. 18 The combination of low body weight and inability to remain hemodynamically stable leads the patient to severe complications and an overall poorer prognosis. These patients are subsequently at a higher risk of pathologies such as refeeding hypophosphatemia, low bone mineral density, and abnormally increased liver enzymes. 18

Typically, patients that deal with eating disorders often fixate on their appearance, especially encompassing an extreme dislike for their body shape and weight. Because these patients hide their weight loss with extra layers of clothing, AN can go unnoticed. 19 It is important that physicians recognize the physical presentation of AN patients so that it can facilitate prompt treatment and recovery. Patients have been found to take many measures to reduce their body weight, including restricting and skipping meals, and certain rituals before mealtime such as cutting their food into very small portions, or spreading the food around the plate as if more food had been consumed. 19 Important questions a physician should ask which can help lead to a certain differential diagnosis include “what have you eaten since yesterday?” and “have you ever eaten more than you wanted to (binge-eating) or used any diet pills or laxatives?” 19 . Being vigilant on the way a patient describes their body weight can help lead the clinician to diagnosing AN. For example, a patient may express extreme dislike for the shape of their body or constantly be analyzing areas of their body in the mirror. Keeping this in mind, a detailed history and physical examination are paramount to detect the unnoticed signs and symptoms of AN. 19

Parameters of nutritional status in anorexia are significantly lower levels of BMI, ideal body weight, lean body, and fat mass. Additionally, AN patients display lower heart rates, blood pressure, body temperatures, and red blood cell and white blood cell count. 17 Taking all this into account, consistent monitoring, and regular follow-up of the AN patient can mitigate harmful or fatal complications of AN.

## Evaluation of AN

When evaluating a patient for AN, physicians must first rule out any emergency medical sequelae that necessitate hospitalization and stabilization. 17 It is necessary to obtain a thorough history and physical examination to rule out any neurological or psychiatric comorbidities through comprehensive evaluation. 20 Many diagnoses can present with similar symptoms to AN; these include hyperthyroidism, malabsorption disorders, diabetes, inflammatory bowel disease, immunodeficiencies, Addison's disease, and some chronic infections. 20 These diagnoses need to be ruled out before confirming the diagnosis of AN. Many psychiatric disorders can coincide with AN, like major depressive disorder (MDD) or OCD. All patients must be screened during their office visits to rule out other psychiatric comorbidities when diagnosing AN. Patient history is the most important tool in diagnosis of AN, as laboratory and physical examination can be normal in the earlier stages of this disorder. 20 A common screening tool used in the primary care setting is the SCOFF questionnaire; although this has a 12.5% false positive rate, it is used as an appropriate screening measure, but not for AN diagnosis. 20 Prior to obtaining the patient's weight, one should assess the patient's hydration status via urinalysis and evaluate their specific gravity, ketone levels, and kidney functions. Recording all initial vital signs such as blood pressure, temperature, pulse, height, weight, and BMI is necessary for evaluating the patients' clinical progression or regression. 17 It is possible that patients may try to falsely raise their weight by wearing multiple layers or hide objects on their person; therefore, the need to obtain their weight in just a hospital gown and underwear may be warranted. 17 Laboratory studies should include a complete metabolic panel, complete blood count, toxicology screen, thyroid levels, liver function tests, and pancreatic enzyme levels. These laboratory measures should be obtained and evaluated to note any electrolyte imbalances or other underlying conditions or complications. 17 A healthcare provider may assess the patient's psychological status by completing comprehensive neuropsychological tests, which are additional measures that comprise evaluating an individual's attention span, psychomotor speed, visuospatial capacity, immediate and long-term memories, recall ability, reaction time, decision-making capacity, etc. There are significant findings of AN such as attentional disengagement, shorter reaction times in copying tasks, poor reaction time, fewer words recalled, worse visuospatial, and immediate memory as well as deficits in decision-making. 21 Patients with chronic AN can present with menstrual irregularities such as amenorrhea. 18 Regularly assessing follicle-stimulating hormone, luteinizing hormone, thyroid-stimulating hormone and prolactin levels can be quite helpful in evaluation of abnormalities and areas to target for management. Furthermore, an electrocardiogram is crucial as AN patients' binge-eating and purging habits can have metabolic changes leading to potentially fatal arrhythmias. Also assessing neuropsychological status can give insight into understanding the possible decline in cognitive function seen in patients enduring AN. 22 Lastly, it is important to acquire bone densitometry so that physicians can evaluate the severity of bone loss in these patients. 18 By being meticulous with evaluation of the AN patient, this can lead to timely diagnosis and treatment, and improved outcomes.

## Differential Diagnosis of Anorexia Nervosa

To improve the outcomes of patients suffering with AN, early and prompt diagnosis, especially at an earlier age, has been shown to be correlated with improved health outcomes. The American Academy of Family Physicians states that patients suffering with AN typically restrict calories or involve themselves in excessive exercise to control their emotional needs. These patients demonstrate an unhealthy and extreme fear of gaining weight. 20 The DSM-V has described two main subtypes of AN: 1) the binge-eating and purging type, and 2) the restrictive type. The person with the former subtype usually demonstrates actions like self-induced vomiting, and laxative or diuretic abuse. The latter subtype is considered in a patient that abstains from the regular binge eating and/or purging for a minimum of 3 months. 23

Majority of times, patients suffering with AN are noticed first by primary care providers. 24 Lebow et al conducted a retrospective clinical cohort study and exemplified the role that primary care physicians can play in the treatment of adolescents trying to recover from restrictive eating disorders, along with facilitating weight gain. 24 This study placed emphasis on the pertinent role that physicians' play in helping to restore AN patients' body weight and improve their overall clinical status by focusing on family-based treatment (FBT) for AN. They found a significant improvement in adolescents' BMI following FBT by primary care physicians. 24 While AN patients may not present to their family physician to help treat their anorexia, patients often present to their family physician for secondary complaints such as amenorrhea or extreme fatigues. Thus, family physicians can potentially combine the treatment of secondary symptoms with FBT, while also focusing on restoring weight . 24

**Fig. 3:**
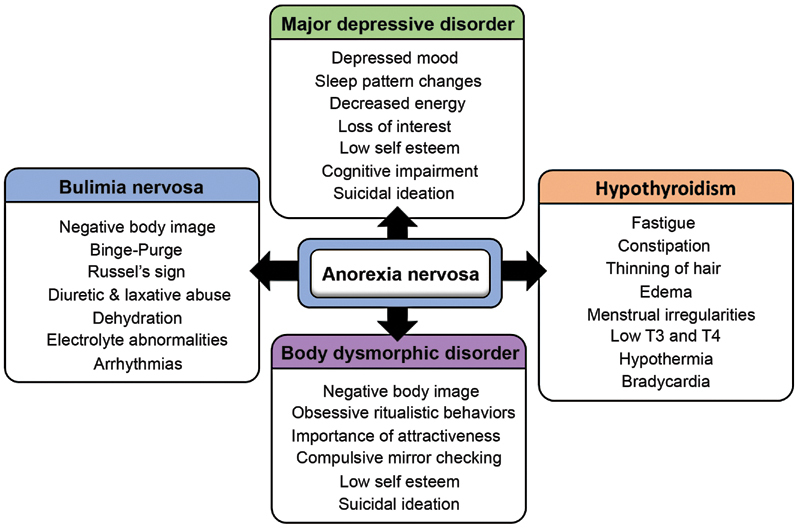
Key similarities between anorexia nervosa and its differential diagnosis. 17 25 26 27 28 29 30 31 32 33 34

AN is a multidimensional disorder that shares significant signs and symptoms with multiple medical conditions. Bulimia nervosa (BN), MDD, body dysmorphic disorder (BDD), and hypothyroidism should be on the differential diagnostic radar due to their shared similarities with AN. 17 25 26 27 Fig. 3 depicts the resemblances between these four disorders. 17 25 26 27 28 29 30 31 32 33 34 Eating-disorder symptoms and psychosis symptoms may coexist. Psychosis may also be considered a severity marker for an eating disorder; on the other hand, varying eating patterns may also be observed as a severity marker for psychosis. There is no consistent sequence in the cooccurrence of the two, and hence, it is essential to look out for these overlapping dimensions of illnesses. 35

Eating disorders such as BN and AN manifest with negative body image, episodes of food binging followed by purging, *Russell's sign* (calluses on the dorsum of the hand, formed by induced vomiting), and the potential of laxative and diuretic abuse. They also share more life-threatening symptoms such as electrolyte abnormalities, severe dehydration, and arrhythmias. 17 30 The most consistent features present in MDD and AN are depressed moods, sleep pattern changes, decreased energy, loss of interest, low self-esteem, cognitive impairment, and suicidal 190 ideations. 17 25 31 AN shares signs and symptoms with endocrinological disorders like hypothyroidisms. Fatigue, constipation, menstrual irregularities, edema, hypothermia, bradycardia, and low thyroid hormones (T3 and T4) are also often discovered in both conditions. 17 26 32 Lastly, AN and BDD are severe psychiatric conditions with comparable personality characteristics. Prevalent features include negative body image, ritualistic behaviors, compulsive mirror checking, and a high value placed on attractiveness. In addition, suicidal ideation is constant in both diagnoses. 27 33 34

Avoidant/restrictive food intake disorder (ARFID) is another differential diagnosis that should be considered when assessing patients with a history of restrictive eating. These patients have either a sensory issue with the appearance, odor, or feel of a specific type of food or they may associate certain types of food to a past traumatic event such as choking or vomiting and avoid whole food groups or avoid eating altogether. Patients who suffer from ARFID may severely restrict the 202 volume of food they consume due to a lack of interest in eating or decreased appetite. 36 While both disorders will cause nutritional deficiencies, decreased body mass, physical and psychosocial disturbances, the reasons for the food aversion are different. ARFID can be differentiated from AN because the food intolerance in patients with ARFID is not due to the fear of gaining weight or because of the pressure on oneself to be aesthetically pleasing, like in AN. 37 Keen history taking and evaluation ate crucial to tell both disorders apart because of the overlapping similarities.

## Treatment of Anorexia Nervosa

According to the American Psychiatric Association, there are three approaches to treating 210 AN: nutritional rehabilitation, psychosocial treatments, and medications. Although the first line of treatment is psychotherapy, a combined approach is superior to either one alone. 38

### Nutritional Rehabilitation

Nutritional rehabilitation is when a patient is given the proper nutrition and calories to help them regain their healthy weight. 39 This initially leads to an increase in lean body mass and eventually increased adipose tissue deposition as the target weight is achieved. 39 Some studies have shown that when hospitalized patients are discharged without reaching their target weight, they relapse and are readmitted to hospitals at a higher rate than their counterparts who attain target weight before discharge. 40 The target weight gain in outpatient scenarios is 0.5 to 1.0 lbs/week, while the step-down programs in which patients are hospitalized for 12 hours a day, 7 days a week have reported a 2 lbs/week weight gain. 41 The most common side effect of such rehabilitation includes RS. RS is defined as an imbalance in the fluid and electrolytes concentration in a patient's body. This results because in a state of starvation the body loses its carbohydrate reserves. Upon reintroduction of carbohydrates, there is an insulin spike that results in potassium moving intracellularly. 42 This complication is much more common in severely malnourished patients and can be avoided with slower feeding, and monitoring of heart rate and rhythm, body weight, and electrolytes especially phosphorus. 39 A study done on 100 adolescent Caucasian females suggested that voluntary supplemental nasogastric nocturnal feeding produced greater and quicker weight gain than oral feedings alone. 43 Rapid weight gain was also observed with high-calorie intake. 44 Therapies such as warming and growth hormone injections have been routinely used for the treatment of patients with AN. However, such therapies have shown no evidence of significant weight gain in these patients. 45 The idea behind warming was based on the fact that AN patients undergoing warming treatment showed a reduction in anxiety, depression, and hyperactivity; hence, it was predicted that decreasing these symptoms would help with weight gain. 45 Warming is usually provided via one of three methods: exposing the patient to a continuous warm environment, using a thermal waistcoat, and/or a sauna bath. 46 A randomized clinical trial done by Birmingham et al involved 21 females with AN who were admitted to the hospital for RS. They were either put into the control arm that received warming via a vest or the treatment arm in which the patients wore the vest, but they were never turned on. This was done to assess if the warming would lead to an increase in the rate of weight gain. Ten patients were allocated to the treatment arm and eleven were allocated to the control arm and followed for 13.6 ± 6.7 years. Both groups wore a heating vest for 3 hours a day for 21 days, but only the experimental group's vests were turned on for that time. The results did not show any significant increase in the rate of weight gain in patients of the treatment group as compared to the controlled group. 47 In a separate double-blind study conducted by de Vos et al, patients were either injected with recombinant human growth hormone or an equal amount of placebo for 28 days every day. This study also showed no significant difference in admission weight, BMI, or caloric intake between the two groups by the end of the study. 48

### Psychosocial Treatments

When it comes to psychosocial treatments, the supporting evidence on efficacy of factors such as psychoeducation, individual and family therapies, and group therapies is inadequate. However, the conclusion that such treatments can be beneficial comes from patient self-reporting and vast clinical experience. 49 In a review of 23 surveys, it was reported that the psychological interventions as well as providing support to the AN patient, understanding their condition, and fostering empathetic relationships were more helpful than the pharmacological therapies focused on weight gain for the treatment of AN. 38 Behavioral programs that focus on individual psychotherapy, family psychotherapy, compassionate nursing approaches, nutritional counseling, and therapies to improve a patient's knowledge and attitude about eating, exercise, and body image have shown good short-term improvements. 50 A review comparing the benefits of behavioral psychotherapy alone versus pharmacological treatment alone found that behavioral psychotherapy results in shorter hospital stays as well as more consistent weight gain among patients with AN, while the same results were not observed with patients treated with medication alone. 50 The strong risk factors for hospital readmission in patients with AN are young age (<15 years), eating attitudes that were markedly abnormal, and a low rate of weight gain while hospitalized. 51 Cognitive behavioral therapy (CBT) has also shown significant efficacy when compared to nutritional counseling in patients with AN. 52 One such study that depicts this was done by Pike et al with 33 patients in the Department of Psychiatry at Columbia University who were either assigned to 1 year of CBT or 1 year of nutritional counseling. 52 The patients assigned to the nutritional counseling group relapsed much earlier and at higher rates than those receiving CBT. The Morgan Russell criteria for “good outcome” were also met by 44% of patients receiving CBT, while only 7% of those receiving nutritional counseling met such criteria. 52 The Morgan Russell criteria are relatively subjective, and patients are either put into good, intermediate, and poor outcome groups. The two main variables that these criteria take into consideration are body weight and the presence or absence of menstruation. 53

When children are diagnosed with AN, the sensitivity of this illness should also take into account the familial beliefs and views as this can be a major setback in their treatment. 54 In adolescents with AN for less than 3 years, family psychotherapy showed more benefits than individual psychotherapy. 54 Individual therapy is when the therapist mainly focuses on the patient dealing with AN. Family psychotherapy involves the family as a whole, along with education and counseling of the family members of the patient suffering with AN. In a follow-up study 5 years later, this observation was reiterated by the results which showed that patients with AN that had an earlier onset in life and a shorter history showed much more benefit with family therapy than that with the individual therapy. 55

### Pharmacological Treatment

AN was formally recognized as an illness over a century ago. 56 Even though much time has passed since that time, the treatment of this condition is as challenging today as it was a century ago. 55 Since patients presenting with AN exhibit symptoms of mood disturbance and OCD, the use of antidepressants has shown some utility in the treatment of AN. 56 The studies on the use of antidepressants in patients with AN for-weight restoration are limited. One such study looked at the addition of fluoxetine with nutritional and psychosocial treatments and found no benefits in respect to rate and the amount of weight gain. 56 57 A 7-week randomized, placebo-controlled, double-blind study of 60 mg of fluoxetine daily in 31 women with AN showed no significant changes in their body weight, measures of eating behavior, or a change in psychological state in patients taking the fluoxetine as opposed to those not on it. 56 A double-blind, placebo-controlled trial done by Kaye et al showed that patients who were put on fluoxetine after weight gain and then followed up after 1 year showed maintenance of weight and lower rates of relapse versus the control arm that was put on placebo. 58 Hence, it can be concluded that fluoxetine has not shown significant results in weight gain; however, it has shown results of weight maintenance in patients who were put on the medication after weight restoration had already been achieved. A separate 5-year two-site study favored CBT over fluoxetine for relapse prevention of AN. 59 An outpatient study observing the weights of underweight adolescent patients treated with psychotherapy plus citalopram versus psychotherapy alone elicited that the weight loss in patients undergoing dual therapy was worse (several kilograms) when compared with psychotherapy alone. 60 The studies on tricyclic antidepressants are very limited. A double-blind controlled study revealed no significant beneficial effects of adding clomipramine to the baseline treatment of patients with AN. 61

Antipsychotics have shown benefits in patients with AN when open label trials were conducted. A study showed that 17 hospitalized patients at Western Psychiatric Institute and Clinic in Pittsburg with AN were subject to an open-label treatment for 6 weeks with olanzapine. These patients showed a reduction in depression, anxiety, core eating disorder symptoms, and a significant weight gain. 62 Thirteen severely ill patients at University of Pisa in Italy with restricting type AN, which is when the patients exercise rigorously and restrict food intake, treated with low-dose haloperidol along with standard therapy revealed significant weight gain and better insight. 63

In regard to managing the complications of AN, supplements such as estrogen–progestins, calcium, and vitamin D are used in practice to reduce osteopenia or osteoporosis but have not shown clinical benefits such as preventing, limiting, or reversing skeletal deterioration. 64 Rather, adequate nutritional rehabilitation during the period of bone growth is the only viable option to possibly reverse bone loss. 65 Six out of forty-four AN patient at Massachusetts General Hospital in a controlled trial treated with estrogen who weighed less than 70% of their healthy body weight showed a 4.0% increase in mean bone density. The matched subjects with comparable body weights (<70% of their healthy body weight) who were not treated with estrogen showed a 20.1% further decrease in their bone density. 66

In conclusion, it is imperative that more research be conducted to create better guidelines for patients diagnosed with AN so that gold standard treatments can be curated and employed rather than physicians basing treatment on their clinical judgment.

## Understanding Treatment Resistance of AN

Both environmental and genetic risk factors paired along with a more susceptible neurobiology are at play in the emerging resistance to treatment in AN and bulimia. 67 Preoccupations with intense fear of weight gain, dietary restrictions, excessive exercise, and how the individual is perceived by society mixed with underlying psychopathology all further add to this issue. Many patients who fall into this cycle of obsessive and restrictive patterns refuse to get treatment. 67 As clinicians, it is essential we recognize the early signs of both eating disorders during the initial primary care appointments. The purpose of this article is to explore possible reasons that contribute to the resistance to treatment in anorexics, including their genetic predisposition, and coexisting psychiatric disorders.

Resistance to the treatment of AN is characterized in a few ways but has no set definition. Treatment-resistant anorexia is identified as a persisting illness of anorexia greater than 7 to 10 years. A study conducted by Broomfield et al classified repeated failed treatments as the second most common criteria as per the published definitions of severe and enduring anorexia nervosa (SE-AN). 68 The DSM-V classifies treatment-resistant anorexia in relation to BMI, symptom severity, additional supervision, and inability to perform daily functions. 68 DSM-V considers a severe BMI to be 15 to 15.99 kg/m ^2^ and an extreme BMI to be less than 15 kg/m ^2^ . 17 DSM-V criteria for inpatient hospitalization for AN are the following: a heart rate of less than 50 beats per minute during the day and less than 45 beats per minute during the night, a systolic blood pressure less than 90 mm Hg, orthostatic changes in pulse greater than 20 beats per minute, a body temperature less than 96 F, body fat less than 10%, a less than 75% ideal body weight, arrhythmia, declining food intake, and unsuccessful outpatient therapy. 17 In-patient treatment programs have shown to be more beneficial and are the best form of treatment available to anorexia patients who have been unsuccessful in outpatient treatment programs. These programs use integrative practices that emphasize the importance of developing a healthy relationship with food, proper eating patterns, and weight gain. 69 In-patient programs also have plans in place to encourage psychological change by educating patients on the benefits of nutritional value, individual as well as group psychotherapy, and regulated meals. 69 Previous studies have categorized readmissions to hospitals and eating disorder clinics to be another basis of identification for treatment resistance among in-patient anorexics. 68 These in-patient programs have a dropout rate ranging from 20 to 51% and a readmission rate ranging from 27 to 42%. 68

Resistance to treatment is a common ordeal among patients with AN. Many anorexics have expressed that they do not want to physically mature into an adult female body in order to avoid separation from their parents and responsibilities. 67 Many of these patients do not have the experience to develop a sense of autonomy, which can lead to low self-esteem, poor personal, and social efficacy. This will often cause anorexic patients to become distressed when faced with life challenges. 67 One theory is that the intense fear of weight gain, dietary restrictions, excessive exercise, distorted perception of oneself distracts the anorexic's mind from life's worrisome events. It is also thought that the cycle of obsessive and restrictive patterns gives anorexics a sense of control and heightens their self-worth. 67 The physical effects of starvation and cognitive decline further add to their treatment resistance. 67

Studies have shown that the function of serotonin and dopamine has been changed in both anorexia and bulimia, which interfere with emotional and behavioral characteristics, and this adds to the mounting resistance to treatment. 67 These changes in serotonin and dopamine are related to increased harm avoidance, which is a count of anxiety and inhibition of behavior. 70 Gamma aminobutyric acid (GABA) is the primary inhibitory neurotransmitter in the brain. Gamma 376 aminobutyric acid receptor subunit gamma-1 (GABRG1) belongs to the family of ligand-gated ionic channels and in part, is what forms the GABA-A receptors. GABA's inhibitory effect is regulated by GABA-A receptors or metabotropic GABA-B receptors. 71 In a study performed by Bloss et al on 1,878 women, the researchers tested 5,151 single-nucleotide polymorphisms (SNPs) in 350 genes for possibly retarding recovery from eating disorders. An intronic SNP in *GABRG1* showed strongest statistical evidence of association with retarding 382 recovery ( *p* = 4.57 × 10 − 6, false discovery rate = 0.0049, odds ratio = 0.55). The same intronic SNP was found to be associated with the anxiety trait ( *p* = 0.049), suggesting a possible genetic mechanism through which this variant may influence the outcome or recovery from eating disorders. 71 Individuals who have SNP on GABRG1 gene, rs17536211, showed decreased chances of developing anxiety, which is one of the most common psychiatric illnesses linked to the treatment resistance in AN. The SNP, rs17536211, also exhibited strong correlation of recovery from eating disorders. 71

Psychiatric comorbidity is another issue that contributes to anorexia treatment noncompliance. 67 According to the U.S. National Survey Replication, 56.2% of anorexics had at least one psychiatric comorbidity. Out of all participants, the most common mental illness was an anxiety related problem, OCD (41%) followed by social phobia (20%.) The anxiety disorders of many of these patients were reported to have started in their youth, long before the onset of their eating disorders. 67 Treatment resistance for eating disorders is generally anticipated by the severity of focal eating disorder psychopathology that develops from an interaction between nature versus nurture, making it vital to assess genetic traits along with environmental risks. 67

## Management of the Treatment-Resistant AN

AN is complex and difficult to treat. Many people with AN are hesitant about seeking help, leading to avoidance and no treatments, even when AN is potentially life-threatening. Their desire to recover from AN may coexist with hesitancy to behavioral change. 70 AN has highly egosyntonic features that make it run a chronic course with an impact on a person's quality of life. Moreover, a large proportion of people do not have access to a specialized AN treatment and for those who do, treatment dropout rates are notably high. 70 Until now AN treatment intervention has focused on addressing weight and changing eating behavior with measuring the outcomes on these variables or the presence or absence of other psychiatric comorbidities. However, people who deal with chronic AN consider quality of life as a more important goal. Hence, one-sized treatment may not fit all in AN. 70 Management of AN requires balance between interventions that focus on physical safety (eating behavior and weight restoration), and those that address psychological distress. There is a discrepancy in what a successful treatment looks like for a person receiving the treatment and the person delivering treatment for AN. Treatment that prioritized both physical safety as well as assisted an individual to grow and develop their identity was positively perceived. Doctors should always address individual differences and determine what works best from person to person. 67

Managing AN, or treatment-resistant AN, also depends on whether weight gain is considered as fear stimulus/cue or an outcome/ consequence. It has been proposed that individualized exposure exercises based on patient-specific configuration will promote better treatment outcomes. The application of exposure therapy without defining which fear-based expectancy one is attempting to violate is nonspecific or contraindicated. 70 Exposure therapy involves approaching the individual with a fear, that is, conditioned stimulus (CS) without the occurrence of the feared outcome, that is, unconditioned stimulus. Over time, the fear (CS) no longer results in fear or anxiety. Inhibitory learning theory suggests that exposure therapy aims to create a new, nonthreat association with the CS, which serves to reduce anxiety and disconfirm the expectation of the feared outcome. In the inhibitory learning model, exposure is designed to maximize the newly learned nonthreat association when the feared stimulus is presented. Exposure therapy may be utilized to inhibit or cease the association between the feared stimulus and the feared outcome. 72

One of the goals of managing a patient with treatment-resistant AN is to provide a treatment of carefully measured intensity along with palliative care. A number of interventions employed by therapists are as follows 68 :

- Giving assurance that weight is not the objective of the management and that the patient can negotiate and collaborate with the whole team to prevent panic and regression.
- Encouraging the patient to explore intellectual pursuits or hobbies that stimulate pleasure and mastery along with cognitive function.
- Encouraging patients to have some kind of social activity to prevent isolation. This can include spending time with a supportive family member or a friend, attending a religious gathering or a support group, or spending time at a favorite place.
- Doing regular physical examination so that the physician and the whole team along with the patient are kept informed about the medical status of the patient, thus, making an informed decision about the supportive steps to be taken further.
- Improving the nutrition in such a way that does not cause weight gain in the patient and the changes sought will be measured against the tolerance of the anxiety that may be triggered.
- Educating the family and relevant others about the psychopathology of patients with AN and provide them solace and support and warn them to not show overt displays of anger or irritation against the patient. 68

### Deep Brain Stimulation

Deep brain stimulation (DBS) is a surgical procedure involving implantation of typically bilateral electrodes in key structures that are believed to drive the pathological activity in AN. 73 Nucleus accumbens has been considered to play a significant role in reward circuitry. 71 A long-term (2-year) follow-up study of DBS of nucleus accumbens in treatment refractory AN patients showed that this procedure is safe and effective in improving BMI as well as psychiatric symptoms. BMI consistently increased over a 6-month follow-up and 2-year follow-up period. Moreover, 12 patients out of the 29 female patients who underwent the procedure achieved a normal BMI (>18.5 kg/m2) by the end of the 2-year follow-up period and 5 patients had more than 30% increase in BMI achieving a lower threshold in a normal range of BMI. Eleven patients had lower than normal BMI at 2-year follow-up period. Cognitive ability evaluated by Mini-Mental State Exam at 6-month and 2-year follow-up was intact. 71

DBS of subcallosal cingulate in treatment-resistant AN (total patients in the study ( *n* )= 16), with a 1-year follow-up period showed significant improvement in mood, anxiety, affective regulation, and BMI. This study also demonstrated how DBS of subcallosal cingulate affects the natural history of chronic AN. 73 Positron emission tomography imaging showed significant changes in glucose metabolism in brain structures implicated in AN at 6 and 12 months compared with baseline indicating that DBS directly affects anorexia-related brain circulatory. 73

Insula, a part of the brain responsible for homeostasis and interoception and the parietal part of the brain, which is responsible for perception of the body, showed changes in glucose metabolism. A number of studies showed that the patients with AN have structural and functional disturbances in anterior cingulate and hypometabolism of the parietal brain. 73 The cingulate plays a role in processing and rewarding value to external stimuli that is affected in AN. The study mentioned above showed that the activity within and adjacent to DBS target, that is, the subcallosal and anterior cingulate was reduced and there was an increase in parietal activity (which is decreased in anorexics). Therefore, DBS can have a broad effect on neural circuits downstream from the DBS target. 73 Additionally, the temporal region responsible for social cognitive behavior also showed increased glucose metabolism. All the above data collected emphasizes that modulation of activity within subcallosal cingulate might lead to long-term changes in cortical circuits that in turn is significant for interpersonal behaviors beneficial in treating anorexics. 73 Another randomized controlled trial (RCT) of DBS of nucleus accumbens and subcallosal cingulate with a 6-month follow-up showed improvement in quality of life (regardless of increase in BMI). This was calculated by the Short-Form 36 questionnaire, increase in BMI, and improvement in AN behavior (reduced use of laxatives, diuretics and decrease in physical activity). 74

### Electroconvulsive Therapy

A systematic review of use of electroconvulsive (ECT) therapy showed that it can be used in refractory AN, particularly those individuals who are having a high-risk behavior or are refusing to eat or drink. 75 Fourteen patients between the ages of 12 and 94 who have eating disorders were included in the study. Of those patients, 13 patients were diagnosed with AN and 1 patient with binge eating disorder. All these patients showed an improvement in eating disorders following ECT. Following the treatment, the median increase in BMI was 3.36. Additionally, 50% of the individuals had normal BMI after ECT as compared 0% before the ECT. 75 In conclusion, ECT can be used in patients as an alternative to forced feeding or forced medicine administration in acute cases or as a supportive treatment along with psychotherapies.

### Psychosocial Therapies in Treatment-Resistant AN

An RCT was performed to evaluate the efficacy of CBT and Specialist Supportive Clinical Management (SSCM) in patients with SE-AN. SSCM involves combining principles of clinical management and supportive psychotherapy. 76 77 Clinical management consists of the assessment of the patients, educating them about the disorder, its outcome with and without treatment. The discussion should be two-way so that we can understand patients' fears and prejudice if any. Supportive psychotherapy consists of demonstration of support, affection, acceptance towards the patient. Acknowledgment of the patient's strength and respecting their defenses allows the clinicians to explore their point of view. 77 There were no differences at the end of treatment between both the treatment groups. However, in patients with CBT group, a 6-month follow-up showed they have better social adjustment. A 12-month follow-up revealed that individuals with CBT have lower eating disorder symptoms and a readiness for recovery when compared to SSCM group. 76

A study showed that enhanced CBT is a transdiagnostic approach. This means that this approach seeks to identify core cognitive-behavioral processes hypothesized to be significant across a range of disorders (AN, BN, and eating disorders not specified) and to develop a treatment that targets these in contrast to CBT which is disorder-specific. 78 In this study, two samples of AN population ( *n* = 99) were given enhanced CBT and results were recorded before the treatment, after the treatment and at 60 weeks of follow-up period. The first step in this study was to increase the patient's motivation to change. This was followed by tackling their eating disorder psychopathology that includes their extreme response to weight and shape, with simultaneous regaining of the weight if the patient is willing to. The last step was to educate the patient to identify the setbacks and develop strategies to overcome them. The result of this study demonstrated an increase in weight along with decrease in the eating disorder psychopathology and other psychiatric features. Two-thirds of both sample populations completed the study and showed high compliance. 78

FBT focuses on educating parents that they are not responsible for AN in their children along with developing strategies to regain weight. Later, parents are guided on how to transition eating and weight control to the adolescent. 79 FBT helps in focusing a healthy relationship between parents and adolescents, whereas adolescent-focused individual therapy (AFT) is based on the fact that patients with AN confuse self-control with their biological needs. It focuses on identifying the patient's emotions and tackling them rather than numbing themselves with starvation. The patients are taught to distinguish their emotional state from their bodily needs and accept their responsibility with food related issues. 79 Another RCT showed efficacy of FBT over AFT in adolescents with AN. FBT was superior in the follow-up period and there was less hospitalization with adolescents who underwent FBT. AFT is a good alternative for patients and families who prefer individualized treatment. 79

### Early Intervention to Prevent Treatment-Resistant AN

Early diagnosis and intervention are significant to prevent SE-AN. AN, if diagnosed early in adolescents and treated with family therapy, shows improved outcomes. A RCT comparing a group of patients with AN who had early intervention versus the late intervention showed that family therapy was superior to individual supportive therapy in terms of remission, weight, and cognitive function in the early intervention group. 80 Another trial at a 4-year follow-up showed a better outcome of family therapy given early in the course of the illness. 80 Single-family therapy is an intervention in which the patient and their family both visit the therapist together. Multifamily therapy works on the same principles as single-family therapy but involves the support of other families that are going through the same situation to overcome the isolation, stigma and maximize the resources at hand. Single-family therapy and multifamily therapy were studied in adolescents with AN. Multifamily therapy showed how bringing families together is potentially powerful for managing AN. 81

One of the obstacles in providing early intervention to patients with AN is the fact that the patients deny anything is wrong with them. We need to minimize the time of untreated symptoms early in the disease to improve the outcome. There should be education in schools and families to recognize early symptoms and prevent delay of treatment. We have to work with families and overcome the barriers of help seeking. 81 Body image programs have been conducted in schools that include activities to improve self-esteem, peer influence, pubertal development, education on maintaining a healthy weight, psychoeducation on eating disorders. A systematic review showed that body image programs conducted in secondary schools have been effective in improving body image and secondary factors relating to body dissatisfaction. Effective programs targeted adolescents aged between 12 and 13 years. 82

## Conclusion

AN is a complex and rapidly progressive illness that can present in various ways. Recognition by a physician includes a thorough history and physical examination, along with pertinent laboratory studies to evaluate any life-threatening electrolyte abnormalities. Physicians must be aware of the overlap of pathologies that can occur with AN for successful and holistic management, and prevention of fatal complications. The treatment of AN is intricate and requires attention to details. Treatment includes multiple modalities such as nutritional rehabilitation along with psychosocial and pharmacological therapies. An astute clinician understands that sometimes multiple treatment modalities can be met with resistance in the patient. An interdisciplinary team of medical professionals should always manage cases of treatment resistance in AN. Not only psychiatrists are mandated to provide quality healthcare to AN treatment resistance, but also mental health counselors, mental health therapists, psychologists, social workers, dietitians, nursing team, and technicians. All the efforts of the collaborated medical team should be coordinated in the support of delivering quality healthcare to help the transition of AN patient to a healthy lifestyle. Achieving a balance between interventions is crucial for a successful outcome of the AN patient.

## Limitations and Recommendations

A clinical approach to management of the AN patients with particular focus on the treatment resistance encounters few limitations including the paucity of reliable double-blinded randomized clinical trials that can provide in-depth detailed strategy for the management of AN treatment resistance. Besides, most of the studies collected and analyzed lacked the deliberation of the cultural component that may influence the responsiveness of AN patients toward therapy options. More randomized clinical trials with larger sample size that contemplate the cultural difference among patients and providers are recommended.
